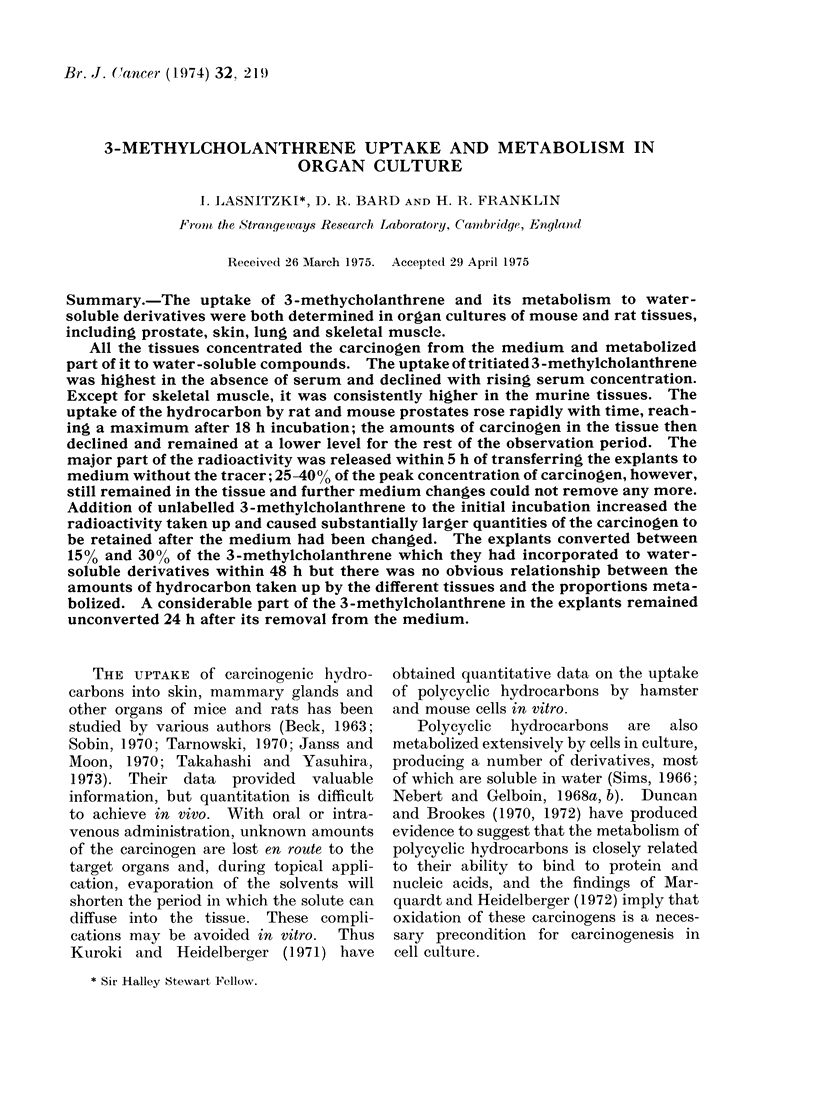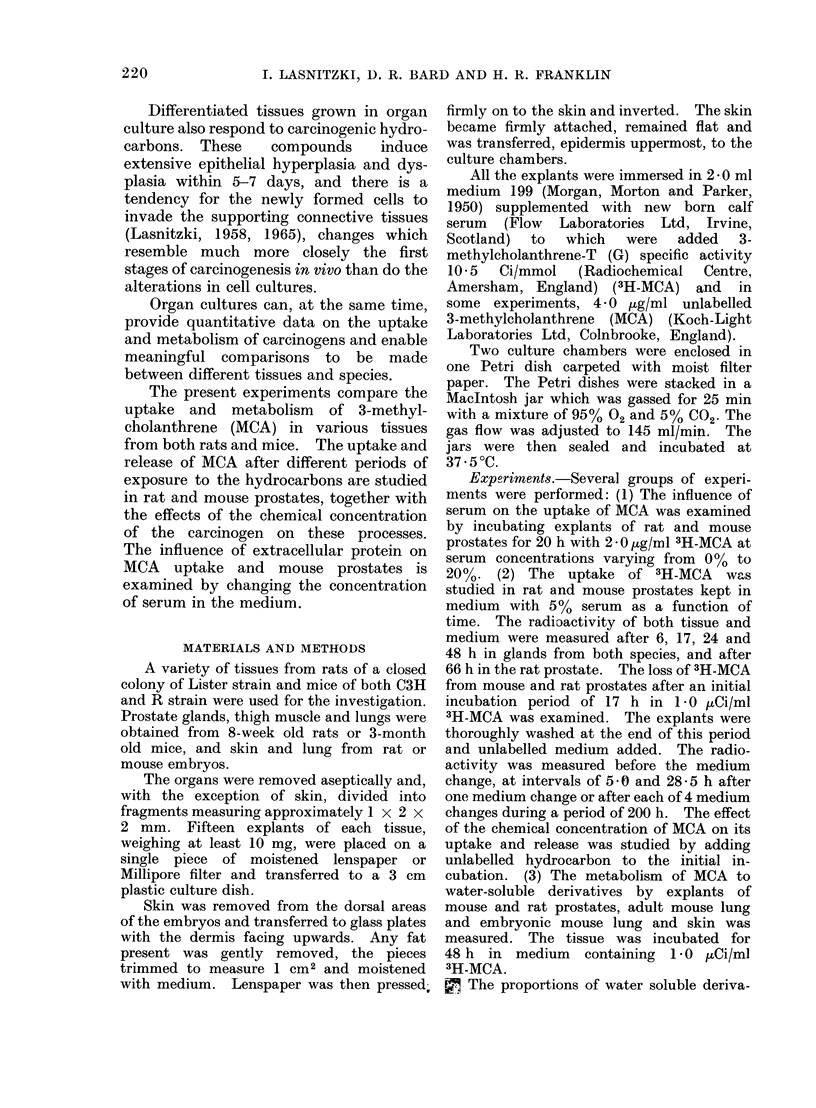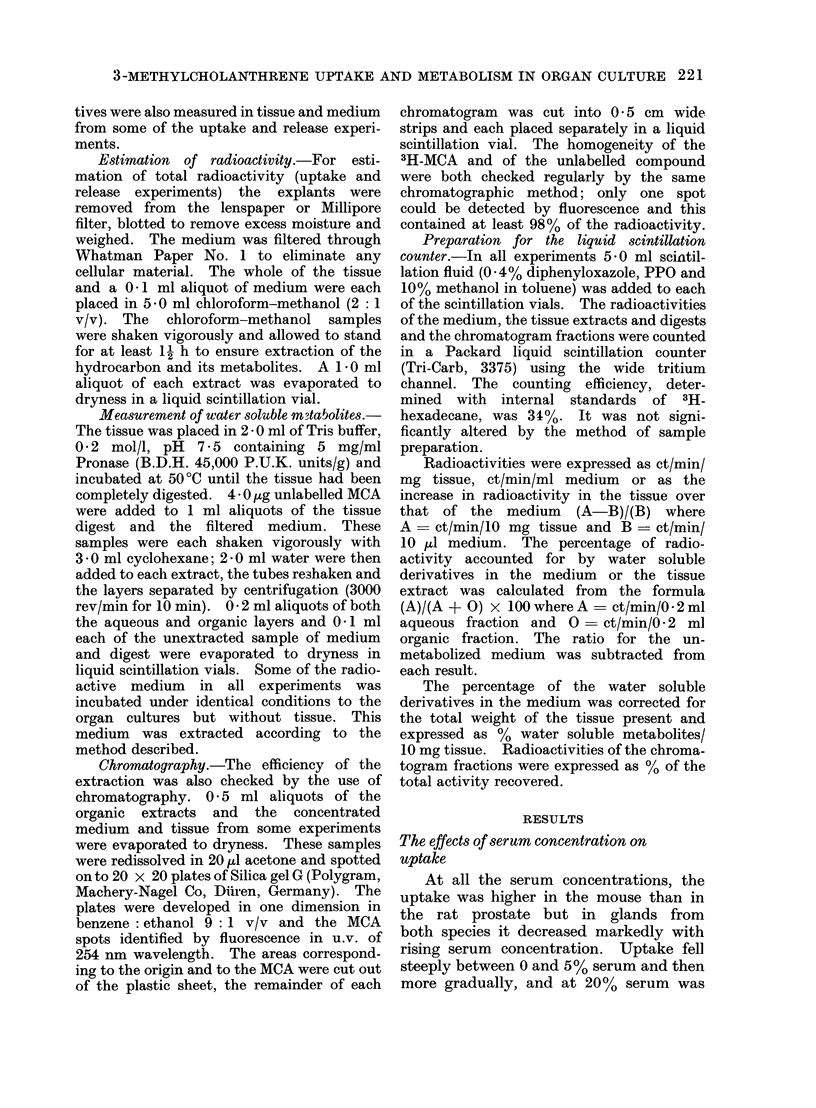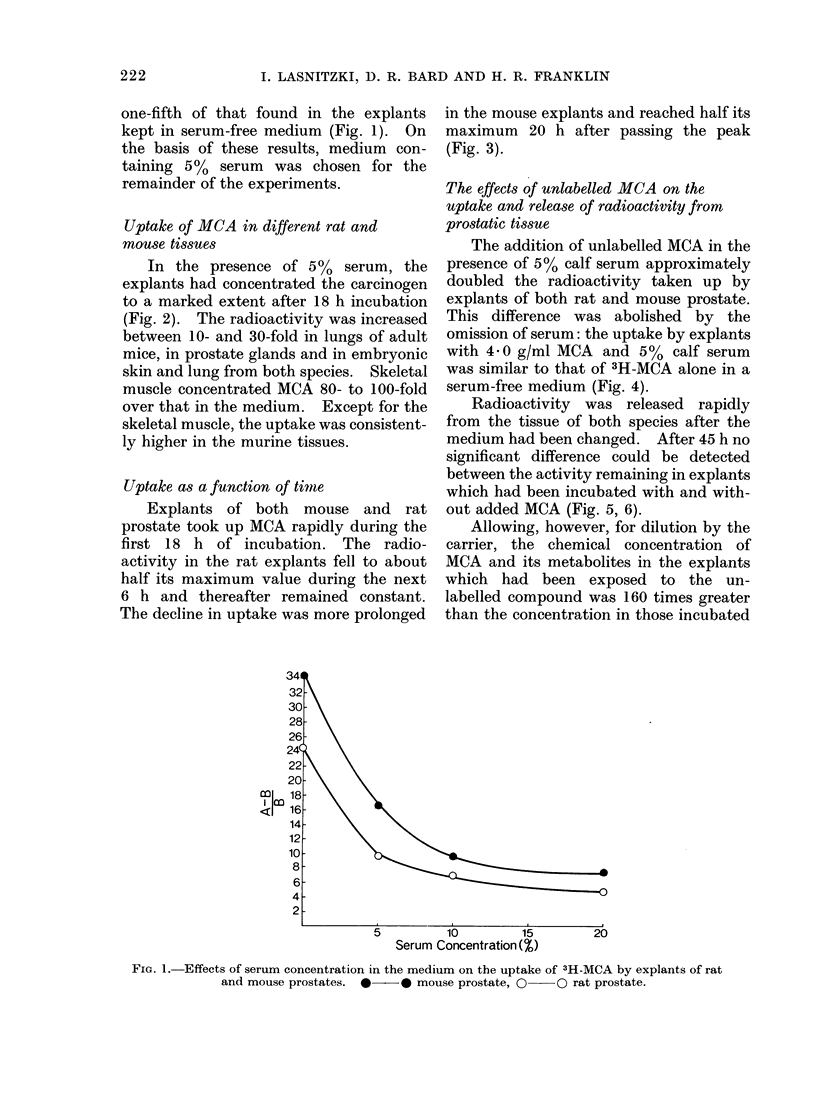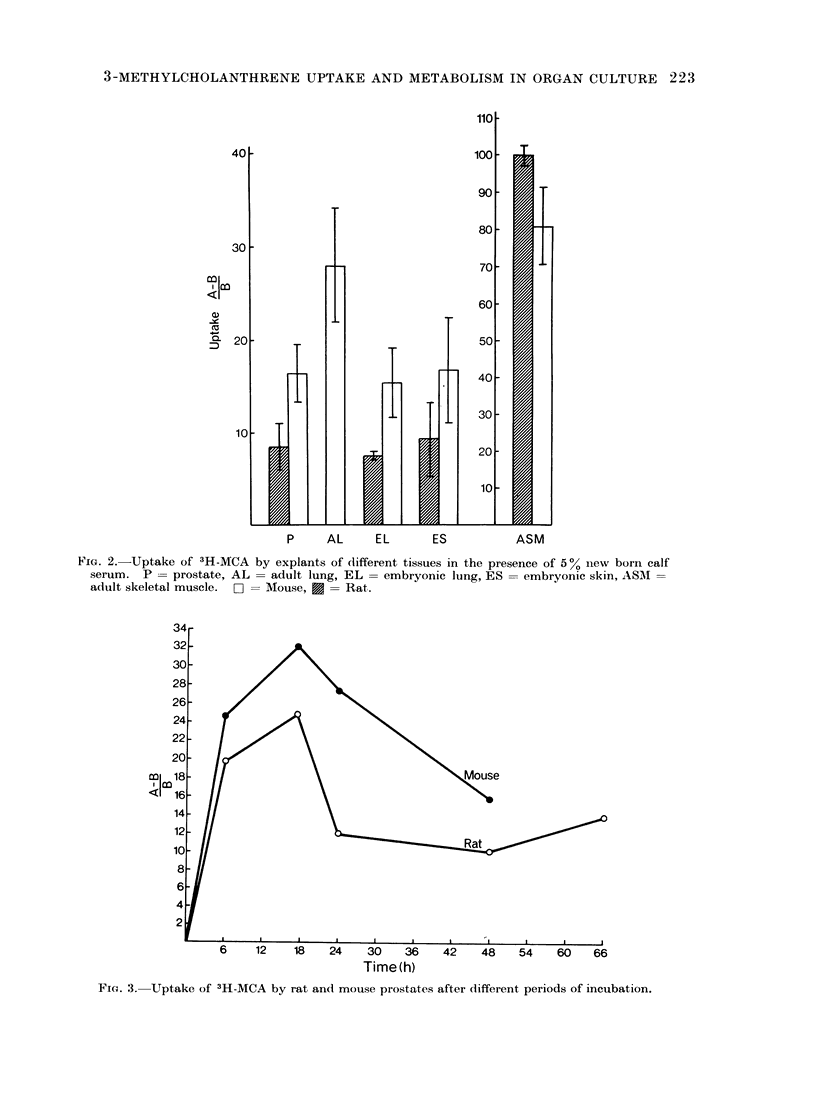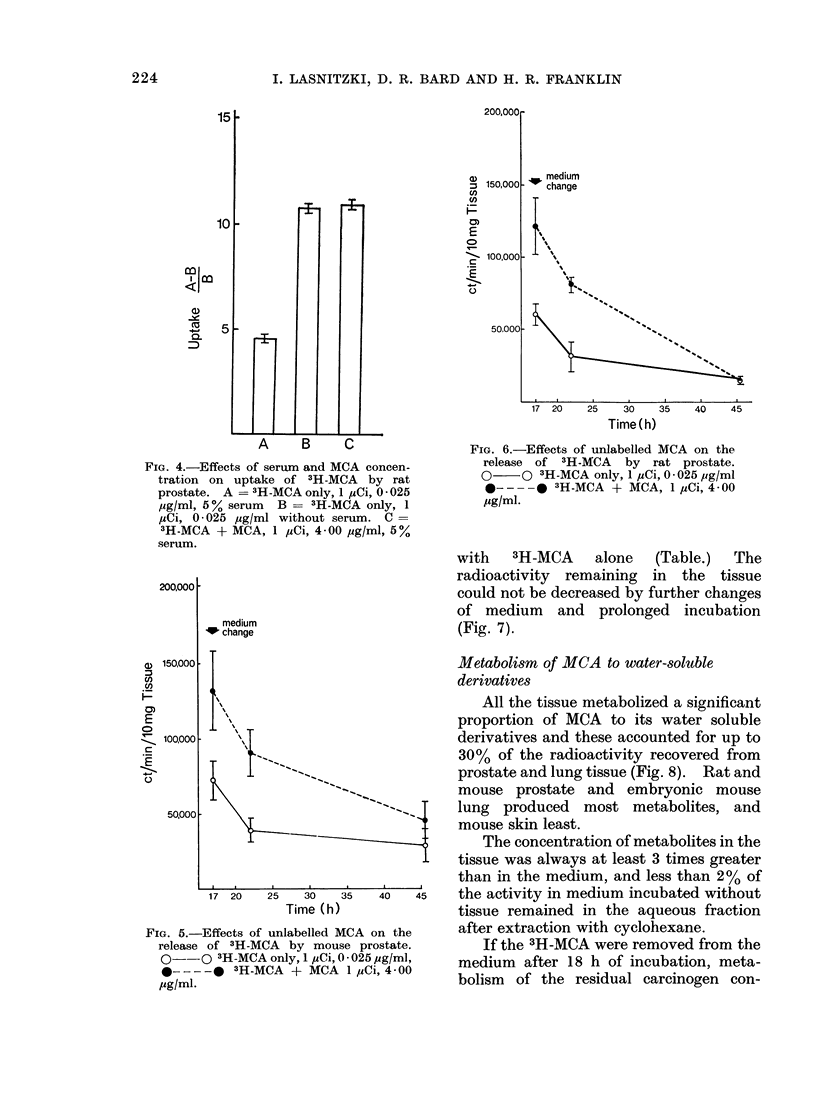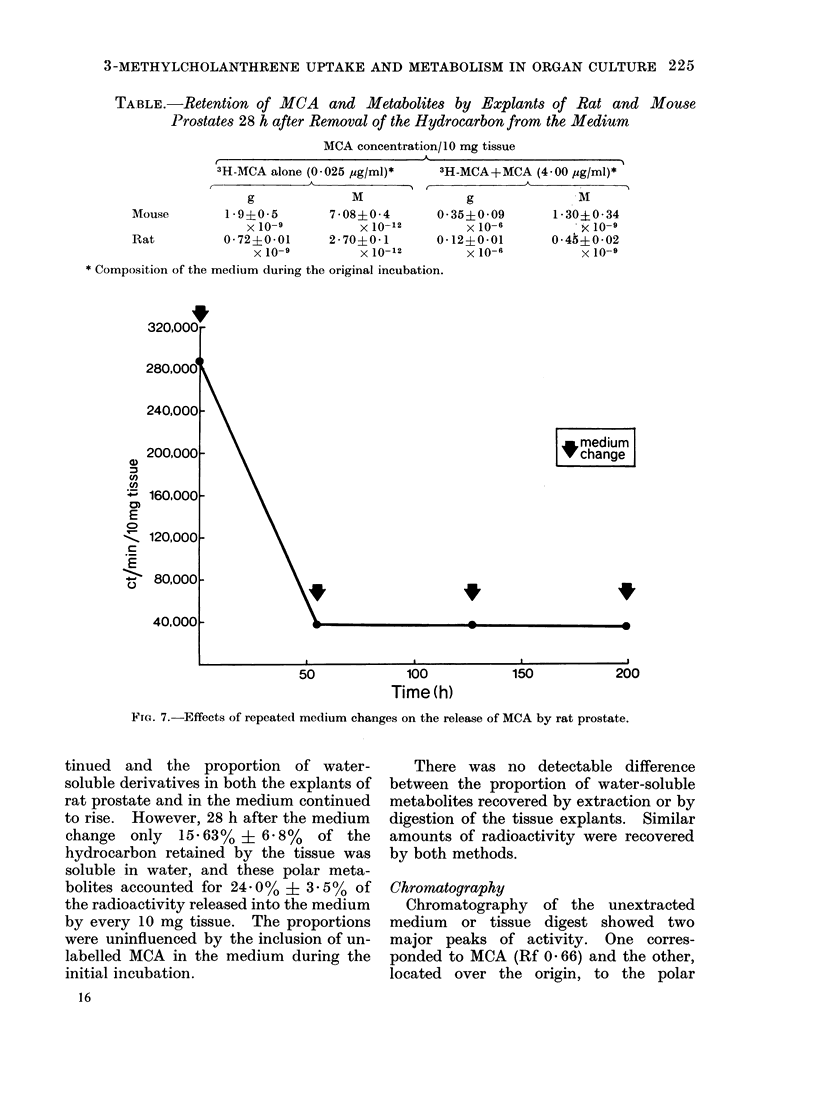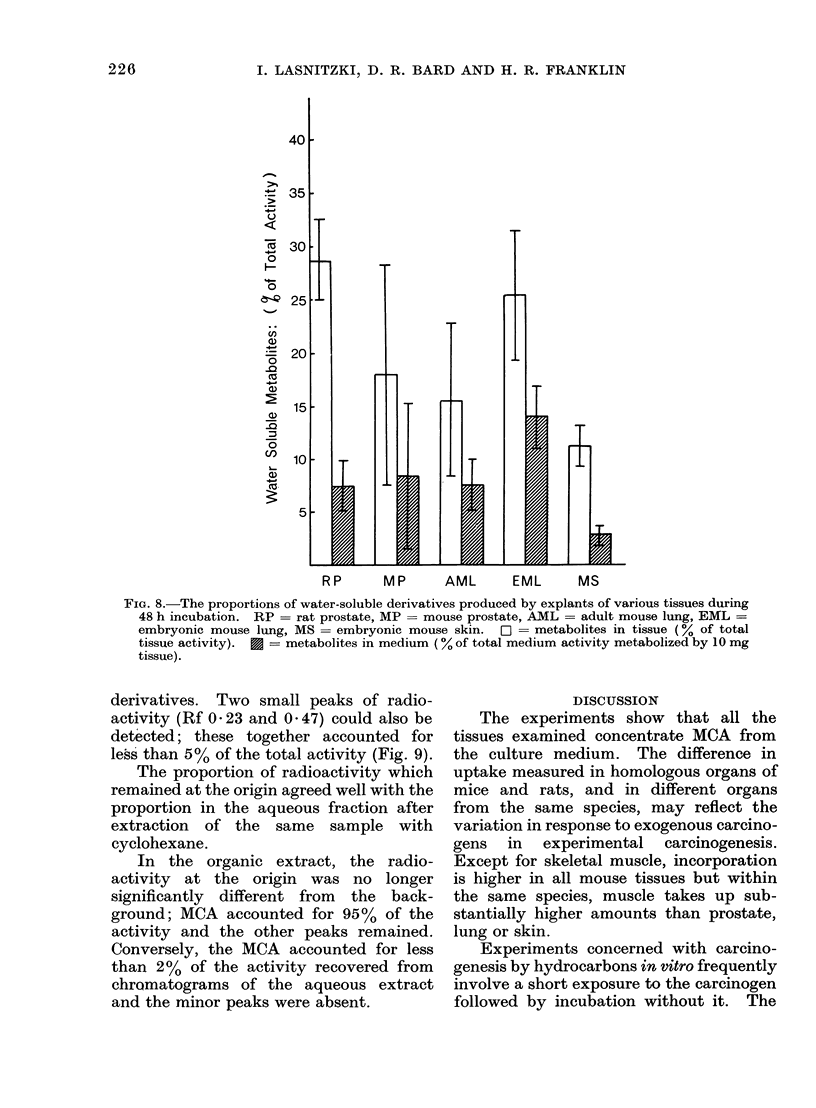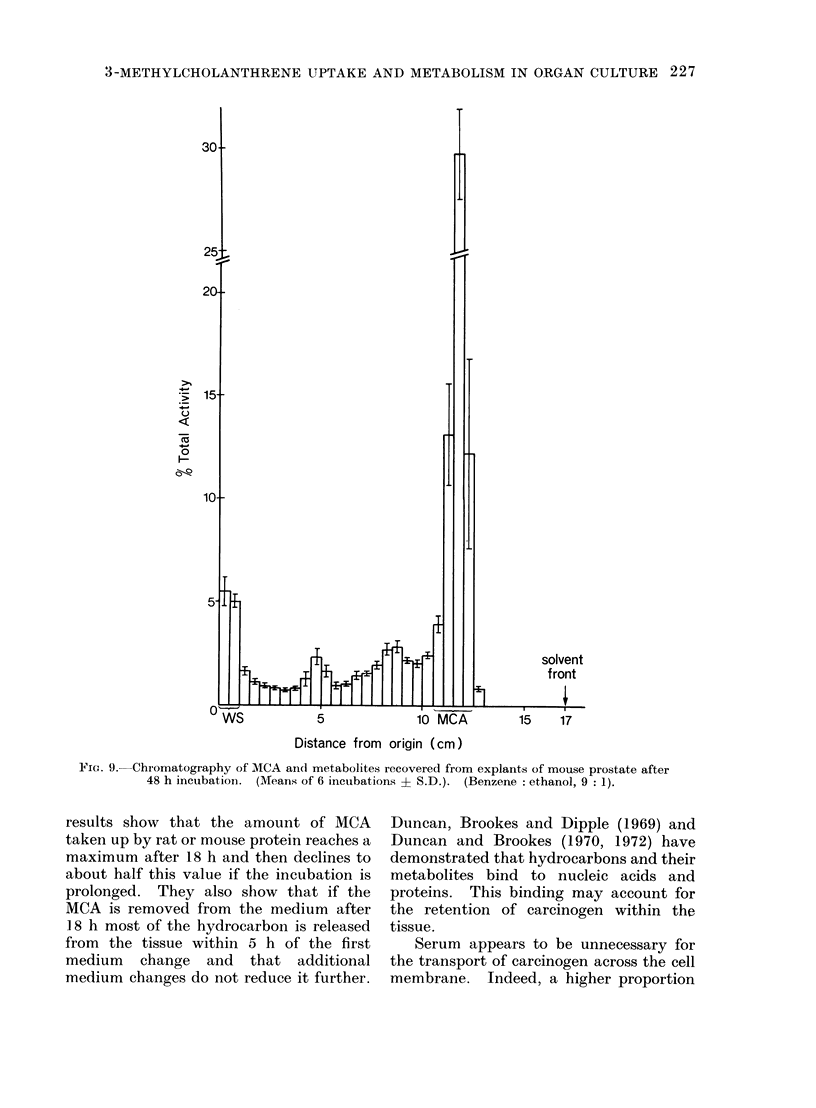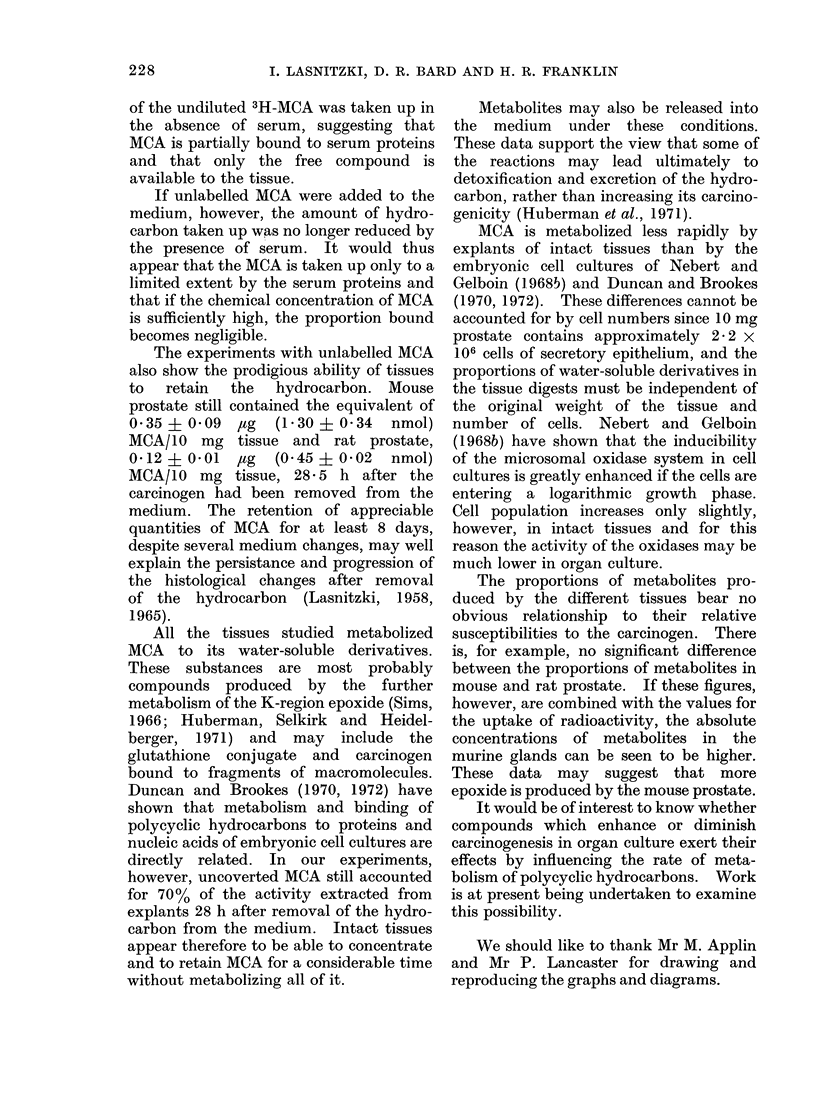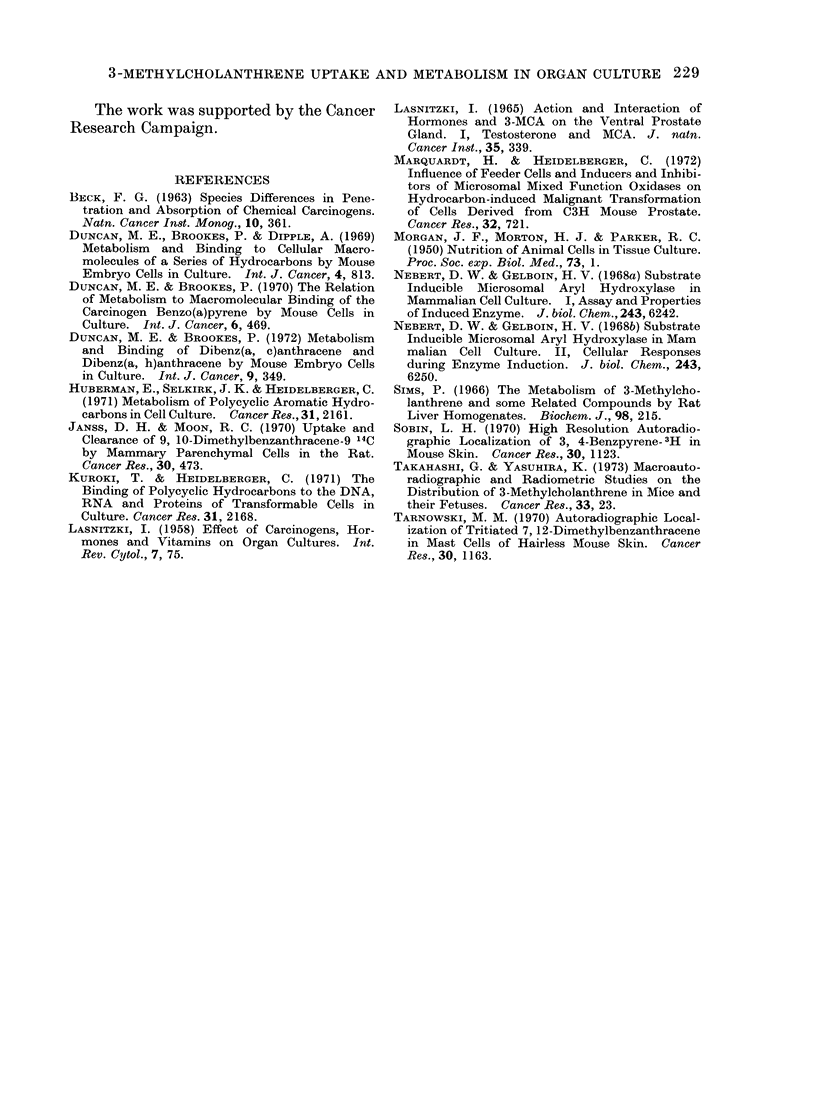# 3-Methylcholanthrene uptake and metabolism in organ culture.

**DOI:** 10.1038/bjc.1975.152

**Published:** 1975-08

**Authors:** I. Lasnitzki, D. R. Bard, H. R. Franklin

## Abstract

The uptake of 3-methycholanthrene and its metabolism to water-soluble derivatives were both determined in organ cultures of mouse and rat tissues, including prostate, skin, lung and skeletal muscle. All the tissues concentrated the carcinogen from the medium and metabolized part of it to water-soluble compounds. The uptake of tritiated 3-methylcholanthrene was highest in the absence of serum and declined with rising serum concentration. Except for skeletal muscle, it was consistently higher in the murine tissues. The uptake of the hydrocarbon by rat and mouse prostates rose rapidly with time, reaching a maximum after 18 h incubation; the amounts of carcinogen in the tissue then declined and remained at a lower level for the rest of the observation period. The major part of the radioactivity was released within 5 h of transferring the explants to medium without the tracer; 25-40% of the peak concentration of carcinogen, however, still remained in the tissue and further medium changes could not remove any more. Addition of unlabelled 3-methylcholanthrene to the initial incubation increased the radioactivity taken up and caused substantially larger quantities of the carcinogen to be retained after the medium had been changed. The explants converted between 15% and 30% of the 3-methylcholanthrene which they had incorporated to water-soluble derivatives within 48 h but there was no obvious relationship between the amounts of hydrocarbon taken up by the different tissues and the proportions metabolized. A considerable part of the 3-methylcholanthrene in the explants remained unconverted 24 h after its removal from the medium.


*Br. J. Cancer* (1974) **32**, 219

### 3-METHYLCHOLANTHRENE UPTAKE AND METABOLISM IN ORGAN CULTURE

I. LASNITZKI*, D. R. BARD AND H. R. FRANKLIN

*From the Strangeways Research Laboratory, Cambridge, England*

Received 26 March 1975. Accepted 29 April 1975

**Summary.—The uptake of 3-methycholanthrene and its metabolism to water-soluble derivatives were both determined in organ cultures of mouse and rat tissues, including prostate, skin, lung and skeletal muscle.**

**All the tissues concentrated the carcinogen from the medium and metabolized part of it to water-soluble compounds. The uptake of tritiated 3-methylcholanthrene was highest in the absence of serum and declined with rising serum concentration. Except for skeletal muscle, it was consistently higher in the murine tissues. The uptake of the hydrocarbon by rat and mouse prostates rose rapidly with time, reaching a maximum after 18 h incubation; the amounts of carcinogen in the tissue then declined and remained at a lower level for the rest of the observation period. The major part of the radioactivity was released within 5 h of transferring the explants to medium without the tracer; 25–40% of the peak concentration of carcinogen, however, still remained in the tissue and further medium changes could not remove any more. Addition of unlabelled 3-methylcholanthrene to the initial incubation increased the radioactivity taken up and caused substantially larger quantities of the carcinogen to be retained after the medium had been changed. The explants converted between 15% and 30% of the 3-methylcholanthrene which they had incorporated to water-soluble derivatives within 48 h but there was no obvious relationship between the amounts of hydrocarbon taken up by the different tissues and the proportions metabolized. A considerable part of the 3-methylcholanthrene in the explants remained unconverted 24 h after its removal from the medium.**

THE UPTAKE of carcinogenic hydrocarbons into skin, mammary glands and other organs of mice and rats has been studied by various authors (Beck, 1963; Sobin, 1970; Tarnowski, 1970; Janss and Moon, 1970; Takahashi and Yasuhira, 1973). Their data provided valuable information, but quantitation is difficult to achieve *in vivo*. With oral or intravenous administration, unknown amounts of the carcinogen are lost *en route* to the target organs and, during topical application, evaporation of the solvents will shorten the period in which the solute can diffuse into the tissue. These complications may be avoided *in vitro*. Thus Kuroki and Heidelberger (1971) have obtained quantitative data on the uptake of polycyclic hydrocarbons by hamster and mouse cells *in vitro*.

Polycyclic hydrocarbons are also metabolized extensively by cells in culture, producing a number of derivatives, most of which are soluble in water (Sims, 1966; Nebert and Gelboin, 1968*a*, *b*). Duncan and Brookes (1970, 1972) have produced evidence to suggest that the metabolism of polycyclic hydrocarbons is closely related to their ability to bind to protein and nucleic acids, and the findings of Marquardt and Heidelberger (1972) imply that oxidation of these carcinogens is a necessary precondition for carcinogenesis in cell culture.

* Sir Halley Stewart Fellow.

Differentiated tissues grown in organ culture also respond to carcinogenic hydrocarbons. These compounds induce extensive epithelial hyperplasia and dysplasia within 5–7 days, and there is a tendency for the newly formed cells to invade the supporting connective tissues (Lasnitzki, 1958, 1965), changes which resemble much more closely the first stages of carcinogenesis *in vivo* than do the alterations in cell cultures.

Organ cultures can, at the same time, provide quantitative data on the uptake and metabolism of carcinogens and enable meaningful comparisons to be made between different tissues and species.

The present experiments compare the uptake and metabolism of 3-methylcholanthrene (MCA) in various tissues from both rats and mice. The uptake and release of MCA after different periods of exposure to the hydrocarbons are studied in rat and mouse prostates, together with the effects of the chemical concentration of the carcinogen on these processes. The influence of extracellular protein on MCA uptake and mouse prostates is examined by changing the concentration of serum in the medium.

### MATERIALS AND METHODS

A variety of tissues from rats of a closed colony of Lister strain and mice of both C3H and R strain were used for the investigation. Prostate glands, thigh muscle and lungs were obtained from 8-week old rats or 3-month old mice, and skin and lung from rat or mouse embryos.

The organs were removed aseptically and, with the exception of skin, divided into fragments measuring approximately 1 $\times$ 2 $\times$ 2 mm. Fifteen explants of each tissue, weighing at least 10 mg, were placed on a single piece of moistened lenspaper or Millipore filter and transferred to a 3 cm plastic culture dish.

Skin was removed from the dorsal areas of the embryos and transferred to glass plates with the dermis facing upwards. Any fat present was gently removed, the pieces trimmed to measure 1 cm$^2$ and moistened with medium. Lenspaper was then pressed firmly on to the skin and inverted. The skin became firmly attached, remained flat and was transferred, epidermis uppermost, to the culture chambers.

All the explants were immersed in 2·0 ml medium 199 (Morgan, Morton and Parker, 1950) supplemented with new born calf serum (Flow Laboratories Ltd, Irvine, Scotland) to which were added 3-methylcholanthrene-T (G) specific activity 10·5 Ci/mmol (Radiochemical Centre, Amersham, England) ($^3$H-MCA) and in some experiments, 4·0 $\mu$g/ml unlabelled 3-methylcholanthrene (MCA) (Koch-Light Laboratories Ltd, Colnbrooke, England).

Two culture chambers were enclosed in one Petri dish carpeted with moist filter paper. The Petri dishes were stacked in a MacIntosh jar which was gassed for 25 min with a mixture of 95% $O_2$ and 5% $CO_2$. The gas flow was adjusted to 145 ml/min. The jars were then sealed and incubated at 37·5 °C.

*Experiments.*—Several groups of experiments were performed: (1) The influence of serum on the uptake of MCA was examined by incubating explants of rat and mouse prostates for 20 h with 2·0 $\mu$g/ml $^3$H-MCA at serum concentrations varying from 0% to 20%. (2) The uptake of $^3$H-MCA was studied in rat and mouse prostates kept in medium with 5% serum as a function of time. The radioactivity of both tissue and medium were measured after 6, 17, 24 and 48 h in glands from both species, and after 66 h in the rat prostate. The loss of $^3$H-MCA from mouse and rat prostates after an initial incubation period of 17 h in 1·0 $\mu$Ci/ml $^3$H-MCA was examined. The explants were thoroughly washed at the end of this period and unlabelled medium added. The radioactivity was measured before the medium change, at intervals of 5·0 and 28·5 h after one medium change or after each of 4 medium changes during a period of 200 h. The effect of the chemical concentration of MCA on its uptake and release was studied by adding unlabelled hydrocarbon to the initial incubation. (3) The metabolism of MCA to water-soluble derivatives by explants of mouse and rat prostates, adult mouse lung and embryonic mouse lung and skin was measured. The tissue was incubated for 48 h in medium containing 1·0 $\mu$Ci/ml $^3$H-MCA.

The proportions of water soluble derivatives were also measured in tissue and medium from some of the uptake and release experiments.

*Estimation of radioactivity.*—For estimation of total radioactivity (uptake and release experiments) the explants were removed from the lenspaper or Millipore filter, blotted to remove excess moisture and weighed. The medium was filtered through Whatman Paper No. 1 to eliminate any cellular material. The whole of the tissue and a 0·1 ml aliquot of medium were each placed in 5·0 ml chloroform–methanol (2 : 1 v/v). The chloroform–methanol samples were shaken vigorously and allowed to stand for at least 1½ h to ensure extraction of the hydrocarbon and its metabolites. A 1·0 ml aliquot of each extract was evaporated to dryness in a liquid scintillation vial.

*Measurement of water soluble metabolites.*—The tissue was placed in 2·0 ml of Tris buffer, 0·2 mol/l, pH 7·5 containing 5 mg/ml Pronase (B.D.H. 45,000 P.U.K. units/g) and incubated at 50 °C until the tissue had been completely digested. 4·0 $\mu$g unlabelled MCA were added to 1 ml aliquots of the tissue digest and the filtered medium. These samples were each shaken vigorously with 3·0 ml cyclohexane; 2·0 ml water were then added to each extract, the tubes reshaken and the layers separated by centrifugation (3000 rev/min for 10 min). 0·2 ml aliquots of both the aqueous and organic layers and 0·1 ml each of the unextracted sample of medium and digest were evaporated to dryness in liquid scintillation vials. Some of the radioactive medium in all experiments was incubated under identical conditions to the organ cultures but without tissue. This medium was extracted according to the method described.

*Chromatography.*—The efficiency of the extraction was also checked by the use of chromatography. 0·5 ml aliquots of the organic extracts and the concentrated medium and tissue from some experiments were evaporated to dryness. These samples were redissolved in 20 $\mu$l acetone and spotted on to 20 × 20 plates of Silica gel G (Polygram, Machery-Nagel Co, Düren, Germany). The plates were developed in one dimension in benzene : ethanol 9 : 1 v/v and the MCA spots identified by fluorescence in u.v. of 254 nm wavelength. The areas corresponding to the origin and to the MCA were cut out of the plastic sheet, the remainder of each chromatogram was cut into 0·5 cm wide strips and each placed separately in a liquid scintillation vial. The homogeneity of the $^3$H-MCA and of the unlabelled compound were both checked regularly by the same chromatographic method; only one spot could be detected by fluorescence and this contained at least 98% of the radioactivity.

*Preparation for the liquid scintillation counter.*—In all experiments 5·0 ml scintillation fluid (0·4% diphenyloxazole, PPO and 10% methanol in toluene) was added to each of the scintillation vials. The radioactivities of the medium, the tissue extracts and digests and the chromatogram fractions were counted in a Packard liquid scintillation counter (Tri-Carb, 3375) using the wide tritium channel. The counting efficiency, determined with internal standards of $^3$H-hexadecane, was 34%. It was not significantly altered by the method of sample preparation.

Radioactivities were expressed as ct/min/mg tissue, ct/min/ml medium or as the increase in radioactivity in the tissue over that of the medium (A—B)/(B) where A = ct/min/10 mg tissue and B = ct/min/10 $\mu$l medium. The percentage of radioactivity accounted for by water soluble derivatives in the medium or the tissue extract was calculated from the formula (A)/(A + O) × 100 where A = ct/min/0·2 ml aqueous fraction and O = ct/min/0·2 ml organic fraction. The ratio for the unmetabolized medium was subtracted from each result.

The percentage of the water soluble derivatives in the medium was corrected for the total weight of the tissue present and expressed as % water soluble metabolites/10 mg tissue. Radioactivities of the chromatogram fractions were expressed as % of the total activity recovered.

### RESULTS

### *The effects of serum concentration on uptake*

At all the serum concentrations, the uptake was higher in the mouse than in the rat prostate but in glands from both species it decreased markedly with rising serum concentration. Uptake fell steeply between 0 and 5% serum and then more gradually, and at 20% serum was one-fifth of that found in the explants kept in serum-free medium (Fig. 1). On the basis of these results, medium containing 5% serum was chosen for the remainder of the experiments.

*Uptake of MCA in different rat and mouse tissues*

In the presence of 5% serum, the explants had concentrated the carcinogen to a marked extent after 18 h incubation (Fig. 2). The radioactivity was increased between 10- and 30-fold in lungs of adult mice, in prostate glands and in embryonic skin and lung from both species. Skeletal muscle concentrated MCA 80- to 100-fold over that in the medium. Except for the skeletal muscle, the uptake was consistently higher in the murine tissues.

*Uptake as a function of time*

Explants of both mouse and rat prostate took up MCA rapidly during the first 18 h of incubation. The radioactivity in the rat explants fell to about half its maximum value during the next 6 h and thereafter remained constant. The decline in uptake was more prolonged in the mouse explants and reached half its maximum 20 h after passing the peak (Fig. 3).

*The effects of unlabelled MCA on the uptake and release of radioactivity from prostatic tissue*

The addition of unlabelled MCA in the presence of 5% calf serum approximately doubled the radioactivity taken up by explants of both rat and mouse prostate. This difference was abolished by the omission of serum: the uptake by explants with 4·0 g/ml MCA and 5% calf serum was similar to that of $^{3}$H-MCA alone in a serum-free medium (Fig. 4).

Radioactivity was released rapidly from the tissue of both species after the medium had been changed. After 45 h no significant difference could be detected between the activity remaining in explants which had been incubated with and without added MCA (Fig. 5, 6).

Allowing, however, for dilution by the carrier, the chemical concentration of MCA and its metabolites in the explants which had been exposed to the unlabelled compound was 160 times greater than the concentration in those incubated

FIG. 1.—Effects of serum concentration in the medium on the uptake of $^{3}$H-MCA by explants of rat and mouse prostates. ●——● mouse prostate, ○——○ rat prostate.

FIG. 2.—Uptake of $^3$H-MCA by explants of different tissues in the presence of 5% new born calf serum. P = prostate, AL = adult lung, EL = embryonic lung, ES = embryonic skin, ASM = adult skeletal muscle. □ = Mouse, ▨ = Rat.

FIG. 3.—Uptake of $^3$H-MCA by rat and mouse prostates after different periods of incubation.

Fig. 4.—Effects of serum and MCA concentration on uptake of $^3$H-MCA by rat prostate. A = $^3$H-MCA only, 1 μCi, 0·025 μg/ml, 5% serum B = $^3$H-MCA only, 1 μCi, 0·025 μg/ml without serum. C = $^3$H-MCA + MCA, 1 μCi, 4·00 μg/ml, 5% serum.

Fig. 5.—Effects of unlabelled MCA on the release of $^3$H-MCA by mouse prostate. ○——○ $^3$H-MCA only, 1 μCi, 0·025 μg/ml, ●- - - -● $^3$H-MCA + MCA 1 μCi, 4·00 μg/ml.

Fig. 6.—Effects of unlabelled MCA on the release of $^3$H-MCA by rat prostate. ○——○ $^3$H-MCA only, 1 μCi, 0·025 μg/ml ●- - - -● $^3$H-MCA + MCA, 1 μCi, 4·00 μg/ml.

with $^3$H-MCA alone (Table.) The radioactivity remaining in the tissue could not be decreased by further changes of medium and prolonged incubation (Fig. 7).

*Metabolism of MCA to water-soluble derivatives*

All the tissue metabolized a significant proportion of MCA to its water soluble derivatives and these accounted for up to 30% of the radioactivity recovered from prostate and lung tissue (Fig. 8). Rat and mouse prostate and embryonic mouse lung produced most metabolites, and mouse skin least.

The concentration of metabolites in the tissue was always at least 3 times greater than in the medium, and less than 2% of the activity in medium incubated without tissue remained in the aqueous fraction after extraction with cyclohexane.

If the $^3$H-MCA were removed from the medium after 18 h of incubation, metabolism of the residual carcinogen con-

TABLE.—*Retention of MCA and Metabolites by Explants of Rat and Mouse Prostates 28 h after Removal of the Hydrocarbon from the Medium*

| | MCA concentration/10 mg tissue | | | |
|---|---|---|---|---|
| | $^3$H-MCA alone (0·025 μg/ml)* | | $^3$H-MCA+MCA (4·00 μg/ml)* | |
| | g | M | g | M |
| Mouse | 1·9±0·5 ×10$^{-9}$ | 7·08±0·4 ×10$^{-12}$ | 0·35±0·09 ×10$^{-6}$ | 1·30±0·34 ×10$^{-9}$ |
| Rat | 0·72±0·01 ×10$^{-9}$ | 2·70±0·1 ×10$^{-12}$ | 0·12±0·01 ×10$^{-6}$ | 0·45±0·02 ×10$^{-9}$ |

* Composition of the medium during the original incubation.

FIG. 7.—Effects of repeated medium changes on the release of MCA by rat prostate.

tinued and the proportion of water-soluble derivatives in both the explants of rat prostate and in the medium continued to rise. However, 28 h after the medium change only 15·63% ± 6·8% of the hydrocarbon retained by the tissue was soluble in water, and these polar metabolites accounted for 24·0% ± 3·5% of the radioactivity released into the medium by every 10 mg tissue. The proportions were uninfluenced by the inclusion of unlabelled MCA in the medium during the initial incubation.

There was no detectable difference between the proportion of water-soluble metabolites recovered by extraction or by digestion of the tissue explants. Similar amounts of radioactivity were recovered by both methods.

*Chromatography*

Chromatography of the unextracted medium or tissue digest showed two major peaks of activity. One corresponded to MCA (Rf 0·66) and the other, located over the origin, to the polar

FIG. 8.—The proportions of water-soluble derivatives produced by explants of various tissues during 48 h incubation. RP = rat prostate, MP = mouse prostate, AML = adult mouse lung, EML = embryonic mouse lung, MS = embryonic mouse skin. □ = metabolites in tissue (% of total tissue activity). ▨ = metabolites in medium (% of total medium activity metabolized by 10 mg tissue).

derivatives. Two small peaks of radioactivity (Rf 0·23 and 0·47) could also be detected; these together accounted for less than 5% of the total activity (Fig. 9).

The proportion of radioactivity which remained at the origin agreed well with the proportion in the aqueous fraction after extraction of the same sample with cyclohexane.

In the organic extract, the radioactivity at the origin was no longer significantly different from the background; MCA accounted for 95% of the activity and the other peaks remained. Conversely, the MCA accounted for less than 2% of the activity recovered from chromatograms of the aqueous extract and the minor peaks were absent.

### DISCUSSION

The experiments show that all the tissues examined concentrate MCA from the culture medium. The difference in uptake measured in homologous organs of mice and rats, and in different organs from the same species, may reflect the variation in response to exogenous carcinogens in experimental carcinogenesis. Except for skeletal muscle, incorporation is higher in all mouse tissues but within the same species, muscle takes up substantially higher amounts than prostate, lung or skin.

Experiments concerned with carcinogenesis by hydrocarbons *in vitro* frequently involve a short exposure to the carcinogen followed by incubation without it. The

FIG. 9.—Chromatography of MCA and metabolites recovered from explants of mouse prostate after 48 h incubation. (Means of 6 incubations ± S.D.). (Benzene : ethanol, 9 : 1).

results show that the amount of MCA taken up by rat or mouse protein reaches a maximum after 18 h and then declines to about half this value if the incubation is prolonged. They also show that if the MCA is removed from the medium after 18 h most of the hydrocarbon is released from the tissue within 5 h of the first medium change and that additional medium changes do not reduce it further. Duncan, Brookes and Dipple (1969) and Duncan and Brookes (1970, 1972) have demonstrated that hydrocarbons and their metabolites bind to nucleic acids and proteins. This binding may account for the retention of carcinogen within the tissue.

Serum appears to be unnecessary for the transport of carcinogen across the cell membrane. Indeed, a higher proportion of the undiluted $^3$H-MCA was taken up in the absence of serum, suggesting that MCA is partially bound to serum proteins and that only the free compound is available to the tissue.

If unlabelled MCA were added to the medium, however, the amount of hydrocarbon taken up was no longer reduced by the presence of serum. It would thus appear that the MCA is taken up only to a limited extent by the serum proteins and that if the chemical concentration of MCA is sufficiently high, the proportion bound becomes negligible.

The experiments with unlabelled MCA also show the prodigious ability of tissues to retain the hydrocarbon. Mouse prostate still contained the equivalent of $0 \cdot 35 \pm 0 \cdot 09$ $\mu$g ($1 \cdot 30 \pm 0 \cdot 34$ nmol) MCA/10 mg tissue and rat prostate, $0 \cdot 12 \pm 0 \cdot 01$ $\mu$g ($0 \cdot 45 \pm 0 \cdot 02$ nmol) MCA/10 mg tissue, 28·5 h after the carcinogen had been removed from the medium. The retention of appreciable quantities of MCA for at least 8 days, despite several medium changes, may well explain the persistance and progression of the histological changes after removal of the hydrocarbon (Lasnitzki, 1958, 1965).

All the tissues studied metabolized MCA to its water-soluble derivatives. These substances are most probably compounds produced by the further metabolism of the K-region epoxide (Sims, 1966; Huberman, Selkirk and Heidelberger, 1971) and may include the glutathione conjugate and carcinogen bound to fragments of macromolecules. Duncan and Brookes (1970, 1972) have shown that metabolism and binding of polycyclic hydrocarbons to proteins and nucleic acids of embryonic cell cultures are directly related. In our experiments, however, uncoverted MCA still accounted for 70% of the activity extracted from explants 28 h after removal of the hydrocarbon from the medium. Intact tissues appear therefore to be able to concentrate and to retain MCA for a considerable time without metabolizing all of it.

Metabolites may also be released into the medium under these conditions. These data support the view that some of the reactions may lead ultimately to detoxification and excretion of the hydrocarbon, rather than increasing its carcinogenicity (Huberman *et al.*, 1971).

MCA is metabolized less rapidly by explants of intact tissues than by the embryonic cell cultures of Nebert and Gelboin (1968*b*) and Duncan and Brookes (1970, 1972). These differences cannot be accounted for by cell numbers since 10 mg prostate contains approximately $2 \cdot 2 \times 10^6$ cells of secretory epithelium, and the proportions of water-soluble derivatives in the tissue digests must be independent of the original weight of the tissue and number of cells. Nebert and Gelboin (1968*b*) have shown that the inducibility of the microsomal oxidase system in cell cultures is greatly enhanced if the cells are entering a logarithmic growth phase. Cell population increases only slightly, however, in intact tissues and for this reason the activity of the oxidases may be much lower in organ culture.

The proportions of metabolites produced by the different tissues bear no obvious relationship to their relative susceptibilities to the carcinogen. There is, for example, no significant difference between the proportions of metabolites in mouse and rat prostate. If these figures, however, are combined with the values for the uptake of radioactivity, the absolute concentrations of metabolites in the murine glands can be seen to be higher. These data may suggest that more epoxide is produced by the mouse prostate.

It would be of interest to know whether compounds which enhance or diminish carcinogenesis in organ culture exert their effects by influencing the rate of metabolism of polycyclic hydrocarbons. Work is at present being undertaken to examine this possibility.

We should like to thank Mr M. Applin and Mr P. Lancaster for drawing and reproducing the graphs and diagrams.

The work was supported by the Cancer Research Campaign.

### REFERENCES

Beck, F. G. (1963) Species Differences in Penetration and Absorption of Chemical Carcinogens. *Natn. Cancer Inst. Monog.*, **10**, 361.

Duncan, M. E., Brookes, P. & Dipple, A. (1969) Metabolism and Binding to Cellular Macromolecules of a Series of Hydrocarbons by Mouse Embryo Cells in Culture. *Int. J. Cancer*, **4**, 813.

Duncan, M. E. & Brookes, P. (1970) The Relation of Metabolism to Macromolecular Binding of the Carcinogen Benzo(a)pyrene by Mouse Cells in Culture. *Int. J. Cancer*, **6**, 469.

Duncan, M. E. & Brookes, P. (1972) Metabolism and Binding of Dibenz(a, c)anthracene and Dibenz(a, h)anthracene by Mouse Embryo Cells in Culture. *Int. J. Cancer*, **9**, 349.

Huberman, E., Selkirk, J. K. & Heidelberger, C. (1971) Metabolism of Polycyclic Aromatic Hydrocarbons in Cell Culture. *Cancer Res.*, **31**, 2161.

Janss, D. H. & Moon, R. C. (1970) Uptake and Clearance of 9, 10-Dimethylbenzanthracene-9 $^{14}C$ by Mammary Parenchymal Cells in the Rat. *Cancer Res.*, **30**, 473.

Kuroki, T. & Heidelberger, C. (1971) The Binding of Polycyclic Hydrocarbons to the DNA, RNA and Proteins of Transformable Cells in Culture. *Cancer Res.* **31**, 2168.

Lasnitzki, I. (1958) Effect of Carcinogens, Hormones and Vitamins on Organ Cultures. *Int. Rev. Cytol.*, **7**, 75.

Lasnitzki, I. (1965) Action and Interaction of Hormones and 3-MCA on the Ventral Prostate Gland. I, Testosterone and MCA. *J. natn. Cancer Inst.*, **35**, 339.

Marquardt, H. & Heidelberger, C. (1972) Influence of Feeder Cells and Inducers and Inhibitors of Microsomal Mixed Function Oxidases on Hydrocarbon-induced Malignant Transformation of Cells Derived from C3H Mouse Prostate. *Cancer Res.*, **32**, 721.

Morgan, J. F., Morton, H. J. & Parker, R. C. (1950) Nutrition of Animal Cells in Tissue Culture. *Proc. Soc. exp. Biol. Med.*, **73**, 1.

Nebert, D. W. & Gelboin, H. V. (1968*a*) Substrate Inducible Microsomal Aryl Hydroxylase in Mammalian Cell Culture. I, Assay and Properties of Induced Enzyme. *J. biol. Chem.*, **243**, 6242.

Nebert, D. W. & Gelboin, H. V. (1968*b*) Substrate Inducible Microsomal Aryl Hydroxylase in Mam malian Cell Culture. II, Cellular Responses during Enzyme Induction. *J. biol. Chem.*, **243**, 6250.

Sims, P. (1966) The Metabolism of 3-Methylcholanthrene and some Related Compounds by Rat Liver Homogenates. *Biochem. J.*, **98**, 215.

Sobin, L. H. (1970) High Resolution Autoradiographic Localization of 3, 4-Benzpyrene-$^{3}H$ in Mouse Skin. *Cancer Res.*, **30**, 1123.

Takahashi, G. & Yasuhira, K. (1973) Macroautoradiographic and Radiometric Studies on the Distribution of 3-Methylcholanthrene in Mice and their Fetuses. *Cancer Res.*, **33**, 23.

Tarnowski, M. M. (1970) Autoradiographic Localization of Tritiated 7, 12-Dimethylbenzanthracene in Mast Cells of Hairless Mouse Skin. *Cancer Res.*, **30**, 1163.